# Contact with child and adolescent psychiatric services among self-harming and suicidal adolescents in the general population: a cross sectional study

**DOI:** 10.1186/1753-2000-8-13

**Published:** 2014-04-17

**Authors:** Anita J Tørmoen, Ingeborg Rossow, Erlend Mork, Lars Mehlum

**Affiliations:** 1 National Centre for Suicide Research and Prevention, Institute of Clinical Medicine, University of Oslo, Sognsvannsveien 21, Building 12, Oslo 0372, Norway; 2 Norwegian Institute for Alcohol and Drug Research, POB 565 Sentrum, Oslo N-0105, Norway

**Keywords:** Self-harm, Adolescents, Help-seeking

## Abstract

**Background:**

Studies have shown that adolescents with a history of both suicide attempts and non-suicidal self-harm report more mental health problems and other psychosocial problems than adolescents who report only one or none of these types of self-harm. The current study aimed to examine the use of child and adolescent psychiatric services by adolescents with both suicide attempts and non-suicidal self-harm, compared to other adolescents, and to assess the psychosocial variables that characterize adolescents with both suicide attempts and non-suicidal self-harm who report contact.

**Methods:**

Data on lifetime self-harm, contact with child and adolescent psychiatric services, and various psychosocial risk factors were collected in a cross-sectional sample (response rate = 92.7%) of 11,440 adolescents aged 14–17 years who participated in a school survey in Oslo, Norway.

**Results:**

Adolescents who reported any self-harm were more likely than other adolescents to have used child and adolescent psychiatric services, with a particularly elevated likelihood among those with both suicide attempts and non-suicidal self-harm (OR = 9.3). This finding remained significant even when controlling for psychosocial variables. In adolescents with both suicide attempts and non–suicidal self-harm, symptoms of depression, eating problems, and the use of illicit drugs were associated with a higher likelihood of contact with child and adolescent psychiatric services, whereas a non-Western immigrant background was associated with a lower likelihood.

**Conclusions:**

In this study, adolescents who reported self-harm were significantly more likely than other adolescents to have used child and adolescent psychiatric services, and adolescents who reported a history of both suicide attempts and non-suicidal self-harm were more likely to have used such services, even after controlling for other psychosocial risk factors. In this high-risk subsample, various psychosocial problems increased the probability of contact with child and adolescent psychiatric services, naturally reflecting the core tasks of the services, confirming that they represents an important area for interventions that aim to reduce self-harming behaviour. Such interventions should include systematic screening for early recognition of self-harming behaviours, and treatment programmes tailored to the needs of teenagers with a positive screen. Possible barriers to receive mental health services for adolescents with immigrant backgrounds should be further explored.

## Background

Self-harm (SH) refers to both suicide attempts (SA) and non-suicidal self-harm (NSSH), and is reported by a high proportion of the adolescent population [1,2] with a recent literature review indicating a mean lifetime prevalence of 17–18% [3].

Self-harming behaviours are closely related to mental disorders or symptoms of mental distress [4-7] and to suicidal ideation [8], and subjects who report self-harm are at increased risk of completed suicide [9-11].

Cutting is the most common method of self-harming behaviour among young people, and the most commonly reported motives are wanting to get relief from intensely unpleasant emotions and wanting to die [12,13]. A distinction is often made between self-harming behaviours with the intent of ending one’s own life (SA) and those without such intent (NSSH) [14,15]. Clinical experiences indicate that many adolescents may alternate between various motives for self-harming behaviour and thus, alternate between SA and NSSH, although we lack systematic longitudinal studies to confirm this. Both clinical and general population studies have shown that adolescents who report both SA and NSSH seem to have more severe psychosocial problems, such as suicidal ideation and mental health problems, psychoactive substance use, and antisocial behaviour, than those who report either SA or NSSH [7,16]. Based on this, it seems natural to assume that adolescents with both SA and NSSH would report a higher use of child and adolescent psychiatric services (CAPS).

However, there is very limited knowledge about the use of services by these at-risk adolescents in general, and their use of specialized psychiatric treatments in particular. Most studies of self-harming adolescents’ contact with help-services have addressed contact with general hospitals, acute and emergency departments, and other health services after episodes of SH. These studies have found that only a small percentage (4–20%) of the adolescents had received such care after an episode of SH [17-21]. Three general population studies on self-harming adolescents and their use of specific mental health services exist [5,22,23], and indicate that less than half of suicidal adolescents had been in contact with treatment services in the previous 12 months. However, the Nock study [5] found lifetime contact with psychiatric specialized care was high, with 86% of the suicidal adolescents reporting contact with some type of mental health specialty. None of these studies has focused specifically on suicide attempts and non-suicidal self-harm.

The aims of this study were to examine the extent to which adolescents who report both SA and NSSH have been in contact with CAPS compared with other adolescents, and to assess which psychosocial variables characterize the adolescents with both SA and NSSH who report using CAPS. Since utilization of mental health services has been found to be strongly associated with the extent and severity of mental health problems [24], we hypothesized that adolescents with both SA and NSSH would be more likely to have been in contact with CAPS compared to others with only NSSH, only SA or to those with no SH. Furthermore, we hypothesized that any differences in such contact between individuals with different types of SH (SA, NSSH, etc.), would largely be explained by differences in the severity of psychosocial problems, such as symptoms of depression, suicidal ideation, eating problems, antisocial behaviour, use of illicit drugs, and heavy drinking episodes. Because the population of Norway is covered by universal health insurance, we hypothesized that socio-economics and an immigrant background would be of little importance for CAPS contact.

## Methods

### Participants and procedures

The present study was based on data from a cross-sectional survey completed by adolescents in the city of Oslo, Norway. All junior and senior high schools (N = 91) in the city were asked to join the study and 75 (82%) of these schools agreed to participate. There was a geographically even distribution of non-attending schools in the city. All pupils in grades 9, 10, and 11 in the study schools were invited to participate, and a strategy for including those who were not attending the particular day of the survey in a second distribution were conducted. The overall response rate was 92.7, increased from 87% on the main day of the survey. The survey was anonymous, hence a license from the Data Inspectorate to process personal sensitive data was not required. Permission from the Ministry of Research and Education, the local school authorities and the school boards were obtained. Study participation was based on informed passive parental consent. The net sample comprised 11,440 participants with a mean age of 15.4 years (range, 14–17 years; 51.2% girls). Pupils completed a comprehensive questionnaire at school during two school hours.

### Measures

The study was designed to allow participants to report episodes of SA and NSSH separately. Self-harming behaviour and suicidal behaviour was assessed with two questions: *“Have you ever taken an overdose of pills or otherwise tried to harm yourself on purpose?”* (“no”, “yes, once”, and “yes, more than once”), a question derived from the CASE study and has been used in several other studies [13,20,25,26]. Suicide attempt was assessed with the question: *“Have you ever tried to kill yourself?”* (“no”, “yes, once”, and “yes, more than once”), which has been used in previous Norwegian school surveys of adolescents [27]. The adolescents were divided into four groups based on their responses to the two questions: 1) no SH, 2) NSSH, 3) SA, and 4) SA + NSSH. We assumed that those who reported an overdose/SH but not SA had no suicidal intent and their SH would belong to the NSSH category, and that those who confirmed SA but not SH had no behaviour belonging to the NSSH category. This is based on the logical assumptions that answering two different questions on self-harm and confirming one, but denying the other, is a way of reporting intent. Previous studies have categorized SH by intent based on a similar methodological approach [4,25].

*Contact with CAPS* was assessed through the question “*Have you ever been in contact with or received help from child and adolescent psychiatric outpatient services?”*

Questions on sociodemographics and psychosocial problems were asked to the adolescents in Norwegian, and measures and questions are used in previous Norwegian studies [7,17,25,28].

*Socio-demographic* variables included information on gender, age, social class, and immigrant background*.* The *social class* variable was an adaptation of Eriksson and Goldthorpe’s social-class categorization (EGP classes) based on the parents’ professional achievement. For the purpose of the current study, the variable was dichotomized into high or low socio-economic status. Dichotomization of information is widely done, and based on findings that parents’ education level is of importance regarding contact with help-services [29].

The adolescents were asked about their own and their parents’ country of birth which formed the basis for a dichotomous variable on immigrant background. Adolescents were categorized as having a *non-Western immigrant background* if the adolescent and/or both of the parents were born in Asia/Africa.

*Current suicidal ideation* was assessed with one question from the Hopkins Symptom Checklist (SCL-90) [30]. This has been found to be a valid approach [31]. Participants were asked whether, during the previous week, they had had thoughts about ending their life, using a scale ranging from 1 to 4 (“not at all”, “a little”, “rather often”, and “very often”). For statistical analysis, this variable was dichotomized into “none or a little” and “rather often or very often”.

*Substance use variables* comprised information about drinking to intoxication and illicit drug use in the 12 preceding months. Because the distribution of these variables was skewed, they were dichotomized into whether or not the respondent had drunk to intoxication, and used other illicit drugs.

*Depressive symptoms* were assessed with six items from the Hopkins Symptom Checklist (SCL-90) [30], using the previous week as a reference period. The shortened version are found to be valid and used in other publications [32] as also the Norwegian translated version [17]. The items were rated on a scale ranging from 1 to 4, with total scores ranging from 6 to 24 and a higher score indicating more depressive symptoms.

*Eating problems* were assessed using an eight-item Norwegian version of the Eating Attitudes Test, found to be a valid version [33,34]. The items were rated on a scale ranging from 0 to 3, with total scores ranging from 0 to 24.

*Antisocial behaviour* was assessed using 19 variables of criminality, rule breaking, and other types of antisocial behaviour that had occurred in the previous 12 months. The variables were derived from a Norwegian version of a questionnaire used originally in the National Youth longitudinal study [35] and from the Olweus Scale for Antisocial Behaviour [36]. Those who responded affirmatively were scored 1 on each of the items, with a sum score from 0 to 19 and a higher score indicating more antisocial behaviour.

*Loneliness* was assessed using the revised UCLA loneliness scale [37] with five items scored on a scale ranging from 1 to 4, with a sum score from 5 to 20 and a higher score indicating more frequent feelings of loneliness.

*Intimate friendship* was examined using the question “*Do you have one close friend you can talk to when you have personal problems?”* The answers were “Yes” or “No”, with the latter labelled “*No intimate friend to talk to*”.

*Self-perceived poor health* was examined using a question on how they perceived their current general health status. The response categories were on a five-point ordinal scale ranging from “very good” to “very poor”. The distribution on this variable was highly skewed, and the responses were dichotomized into “good self-perceived health” versus “poor or very poor self-perceived health”.

### Analytic strategy and statistical analyses

In the first step we compared the proportion who reported contact with CAPS between three SH-groups; those who reported both SA and NSSH; those who reported either SA or NSSH; and those who reported no SH. Two hypotheses were put to test. First, that adolescents with both SA and NSSH would be more likely to have been in contact with CAPS than other SH groups and second, that any differences in such contact, would largely be explained by differences in the severity of psychosocial problems. We therefore analysed the bivariate association between SH groups and CAPS contact, and we further analysed the bivariate associations between these two variables on the one hand and indicators of psychosocial problems on the other. To address the second hypothesis, we compared the results of bi-variate and multi-variate analyses where CAPS contact was regressed on SH groups. All these analyses were conducted using the entire data set (n = 10976). The rationale for combining those who reported either SA or NSSH, was that we in a previous study using the same data set [7] found that these two SH groups were similar with respect to psycho-social problems.

In the next step we assessed in bi-variate and multi-variate models which psychosocial variables characterized the use of CAPS within a sub-sample, which comprised those with both SA and NSSH (n = 490).

All statistical analyses were conducted using SPSS, version 21SPSS (Inc., Chicago, Illinois). In the bi-variate analyses, we first applied cross-tables and chi-square tests for categorical variables, and analysis of variance and F-tests for continuous variables. Logistic regression models were employed to estimate unadjusted and adjusted odds ratios for predictors of CAPS contact in both steps of the analyses. In the multivariate logistic regression analyses, we applied a stepwise procedure based on model-fit criteria (log likelihood ratio). The covariates considered for inclusion in the multivariate models had demonstrated a bivariate association (p < 0.20) with the outcome variable (CAPS contact). Missing data were excluded list wise.

## Results

Respondents who answered questions on self-harming behaviour and contact with CAPS were categorised into NoSH (n = 8857), NSSH or SA (n = 892) and NSSH + SA (n = 490). Contact with CAPS was significantly related to SH, and adolescent with both SA and NSSH were most likely to report contact. Thirty-four per cent (n = 168) of them reported such contact, 17.6% (n = 157) of those with SA or NSSH reported such contact, and of those with no SH, 5.3% (n =467) (χ^2^ = 680.90 (2), p < 0.00) reported to have had CAPS contact.

As Table 1 shows, there were significant variations between SH groups on selected demographic and psychosocial variables. Behavioural and mental health problems were more often reported by those with SH, and particularly so by those with both SA + NSSH. Correspondingly, these problems were more often reported among those with CAPS contact compared to those without.

**Table 1:** Psychosocial variables according to self-harm group and contact with child and adolescent psychiatric services

**Psychosocial variables**	**No SH (n = 9461)**	**NSSH or SA (n = 969)**	**NSSH + SA (n = 490)**	**Chi sq**	**p**	**No CAPS**	**CAPS**	**Chi sq**	**p**
Females, %	48.7	71.6	71.7	273.8	0.001	51.5	54.4	2.4	0.121
Non-Western immigrant background, %	22.5	19.4	24.6	11.5	0.021	21.7	14.9	21.9	0.001
Low socioeconomic status, %	62.9	65.3	70.8	12.6	0.002	62.9	68.1	7.4	0.006
Illicit drug use, %	8.7	20.7	39.2	576.7	0.001	9.4	28.2	272.6	0.001
Been Drunk, %	11.5	18.7	28.8	159.3	0.001	12.1	23.0	75.6	0.001
Current suicidal ideation, %	3.6	24.7	55.5	2221.5	0.001	6.4	24.0	316.0	0.001
**F-test**					**F-test**				
Depressive symptoms, mean (SD)	11.3 (4.1)	15.4 (4.6)	17.8 (4.7)	900.9	0.001	11.7 (4.3)	15.1 (5.2)	410.3	0.001
Eating problems, mean (SD)	5.6 (4.7)	8.7 (5.5)	10.3 (6.1)	351.3	0.001	5.9 (4.9)	8.4 (6.2)	178.0	0.001
Antisocial problems, mean (SD)	2.7 (3.3)	4.2 (4.1)	5.9 (5.3)	277.8	0.001	2.8 (3.3)	5.0 (5.1)	344.3	0.001

The likelihood of having had CAPS contact was higher for those with SH than those with no SH; unadjusted odds ratio (OR) was 4.1 for CAPS contact in the NSSH or SA only group (95% CI = 3.3, 5.3), and 9.4 in the SA + NSSH group (95% CI = 7.6, 11.6). Whether this association could be attributed to demographic and psycho-social factors was then assessed by estimating multi-variate logistic regression models, adjusting for demographic and psychosocial variables. These analyses indicated that the association between SH groups and CAPS contact could only be partially explained by these other factors (gender, non-Western immigrant background, i.e. from Asia/Africa, illicit drug use, being intoxicated, current suicidal ideation, depressive symptoms, eating problems, and antisocial problems). While the un-adjusted ORs for NSSH or SA only was 4.1 (95% CI = 3.3,5.3) and for SA + NSSH was 9.4 (95% Confidence interval, CI = 7.6, 11.6) (no SH was the reference group), the adjusted ORs were2.28 (95% CI 1.80-2.89) and 3.6 (95% CI = 2.8, 4.8), respectively. Overall, contact with CAPS were related to self-harm group, as the demographic and psychosocial variables could explain much of the variance in CAPS contact, but not all. Odds Ratios for all the groups were reduced by controlling for psychosocial and demographic variables, but still heightened and significantly higher in the group with both NSSH and SA.

In the next step, we explored which variables could explain the likelihood of CAPS contact within the subsample of those with both SA and NSSH (n = 490). Bi-variate analyses showed that non-western background, illicit drug use, loneliness, self-perceived poor health, depressive symptoms, eating problems, and anti-social behaviours all were statistically significantly associated with CAPS contact. However, in the multi-variate logistic regression model, the variables that best contributed to explaining the likelihood of CAPS contact in this group were having a non-western immigrant background, illicit drug use, depressive symptoms, and eating problems. Adolescents with a non-western immigrant background were significantly less likely to have had CAPS contact, while those who reported illicit drug use were more likely to have had CAPS contact. Moreover, the likelihood of having had CAPS contact increased with depression symptoms scores and with eating problems scores (Table 2).

**Table 2:** Summary of adjusted and unadjusted logistic regression analysis for variables associated with CAPS contact in adolescents with NSSH and SA(n = 490)

**Psychosocial variables**	**CAPS contact n = 168**	**No CAPS contact n = 322**	**Unadjusted odds ratio (CI 95)**	**Adjusted odds ratio (CI 95)**^**a**^
Age 14–17, mean (SD)	15.5 (0.8)	15.4 (0.9)	1.12 (0.9-1.4)	
Female, n (%)	119 (70.8)	236 (73.3)	1.90 (0.8-1.7)	
Non-western immigrant background, n (%)	26 (16.0)	87 (28.2)	0.49 (0.3-0.8)*	0.34 (0.2-0.6)*
Low socioeconomic status, n (%)	102 (73.4)	170 (68.8)	1.25 (0.6-1.3)	
Illicit drug use, n (%)	90 (53.6)	78 (46.4)	1.72 (1.1-2.5)*	1.99 (1.1-3.0)*
Been drunk, n (%)	52 (26.6)	82 (33.5)	1.43 (0.8-2.9)	
Current suicidal ideation, n (%)	94 (58.4)	169 (53.1)	1.24 (1.2-2.5)	
Loneliness, n (%)	57 (35.8)	95 (31.6)	1.21 (1.1-2.7)*	
No intimate friend talk to, n (%)	115 (72.4)	210 (72.2)	1.29 (0.8-1.8)	
Self- perceived poor health, n (%)	44 (26.5)	54 (17.0)	1.76 (1.1-2.8)*	
Depressive symptoms, mean (SD)	18.8 (4.3)	17.1 (4.8)	1.08 (1.0-1.1)**	1,08 (1.0-1.1)*
Eating problems, mean (SD)	11.4 (6.3)	9.5 (5.9)	1.05 (1.0-1.1)**	1.04 (1.0-1.1)*
Antisocial behaviours, mean (SD)	6.5 (5.4)	5.4 (4.9)	1.05 (1.0-1.1)**	

*Significant at p < .05. **Significant at p < .01.

^a^Stepwise forward model fit criteria procedure, variables with p < .20 entered in multivariate analyses.

(n = 490).

More than half of those with both SA and NSSH reported current suicidal ideation, but there was no association between current suicidal ideation and contact with CAPS.

## Discussion

We found that a third of adolescents who reported both SA and NSSH had been in contact with CAPS. This proportion was higher than among adolescents in the other SH groups and among those who had not self-harmed. This difference in CAPS contact remained between the groups even when controlling for differences in demographic variables, substance use, mental health, and behavioural problems. Thus, psychosocial and demographic variables explains some, but not all of the differences in CAPS contact between self-harm groups Within the SA + NSSH group, we found that the likelihood of CAPS contact was significantly higher among those using illicit drugs, and it increased with depressive symptoms scores and with eating problems scores. The likelihood was significantly lower among those with a non-Western immigrant background.

Although previous studies have not focused specifically on specialized mental health services, they have generally shown that self-harming adolescents receive little or no professional help after SH in most cases [17-21,38,39]. In our study focusing on CAPS contact, we found the same pattern; most self-harming adolescents report no such contact. Nevertheless, in our sample, contact with CAPS was reported twice as often by those who had both SA and NSSH than by those who reported SA or NSSH only Similar results has been found in a smaller clinical sample [40]. This difference could be associated with the higher burden of mental health and behavioural problems in the SA + NSSH group, in accordance with our hypothesis that variations in psychosocial problems would partly explain variations in CAPS contact. It is possible that these problems *per se*, rather than the SH behaviour, led to the CAPS contact. In line with this, a recent study [5] revealed that the proportion of adolescents who had received mental health treatment increased with the severity of their suicidal behaviour (i.e., from ‘suicidal ideation’ to ‘having a plan to commit suicide’), and the authors suggested that adolescents who have problems clinically severe enough to become suicidal more typically enter treatment before the onset of suicidal behaviour.

Not only at-risk adolescents’ need for mental health services, but also barriers to seek contact with those services may explain variations in their use of such services. Adolescents’ access to mental health services strongly depends on the parents, teachers, and other adult’s ability to recognize, and to respond to the adolescent’s needs, as well as the adolescents’ own knowledge and perception of their problems [41,42]. A recent review showed that among adults with suicidal behaviour [43], individual factors such as the persons own perception of little or no treatment needs, and the wish to handle problems alone, could explain why the person refrained from seeking help. These may be obstacles to seeking treatment among adolescents as well. However, these questions are not addressed in this study.

In the present sample, having a non-Western immigrant background was associated with a significantly lower likelihood of having had CAPS contact in adolescents with both SA and NSSH. Previous studies from North America indicate that ethnic minority youths are less likely to receive mental health care [22,44,45], a finding that has been attributed to low income and insufficient health insurance among ethnic minority groups. Because all citizens in Norway are covered by universal health insurance, the low income in minority groups cannot fully explain the observed differences in CAPS contact in our sample. Other factors that may explain these differences include language barriers, and culturally conditioned differences in how mental health problems and psychiatric treatment are viewed. As a comment on the process of help-seeking, Cauce and co-workers (2002) point out that various studies have found ethnic group differences in mental health-care utilization [46]. They argue that there are three identifiable stages along the help-seeking pathway; problem recognition, decision to seek help and service selection, and conclude that in particular among ethnic minorities, obstacles in all these stages can be found.

Various psychosocial problems constitute the core tasks for CAPS and we found, accordingly, that depressive symptoms, eating problems, and substance use were associated with increased probability of treatment contact. One can argue that these symptoms may more easily be detected by others, and often lead to distress and worry among those in contact with the adolescents. As noted earlier, adolescents are quite dependent on others recognising and responding to their problems, and this could partly explain their increased contact. It is also of interest that these symptoms are among the most frequent reasons for referrals to outpatient and in-patient psychiatric treatment in Norway [47].

### Strengths and limitations

Among the strengths of this survey study was the large sample derived from the general population of adolescents and a very high response rate strengthening the external validity of the findings. Even those who did not attend on the day set for the survey were given the chance to respond later, an important strategy to prevent important information from a group who in earlier research are found to be prone to “at risk” behaviour [48]. As in all cross-sectional surveys, the design prevents inferences about causal relationships. Furthermore, retrospective reporting will always generate possibilities of recall bias. As in most large-scale population studies, most of our observations rely on one or a few variables to cover each dimension and this could potentially inflate both false-negative and false-positive responses. We had no information on the temporal relationship between SH and the time of contact with CAPS. Moreover, both SH and contact with CAPS referred to lifetime experiences, whereas the psychosocial characteristics that were assessed pertained to experiences in the past year or past week. Internal barriers to service use and other factors of potential importance to the association between SH and contact with CAPS were not available for assessment in this study.

### Implications

The results of this study may be useful for making improvements to health services for adolescents living in urban areas with ethnically and socio-economically diverse backgrounds. Knowing that young people are hesitant to seek professional help, lowering the threshold for contact with CAPS for suicidal and self-harming youths is of importance. Untreated mental illness is a prevalent phenomenon worldwide [49], representing prolonged individual suffering. The receipt of help is especially important for young individuals who self-harm, both to curb their increased risk for completed suicide later in life [50] and to prevent severe distress in the developmentally important stage of adolescence.

Our study suggests that contact with CAPS is not only related to psychiatric symptoms and problem behaviours such as depression, illicit drug use, and eating disorders, but also, to a large extent, self-harming behaviour. Strategies for early and effective identification of self-harm are therefore needed. Clinical experiences have shown that adolescents who self-harm commonly conceal their self-harming behaviour [51], and this should be counteracted by careful assessment and screening procedures. Interestingly, Nock et al. [5] found that adolescents typically enter treatment before, rather than after, the onset of SH. Thus, mental health service providers have important opportunities and challenges in preventing SH and to intervene and counteract the consequences of SH.

This study represents an area of research where knowledge is very limited, especially outside the North American context. Further general population-based studies are needed to gain more knowledge about the treatment needs of self-harming adolescents in non-clinical populations. Of particular importance is the study of possible barriers to receiving treatment among minority youths. Interviews with adolescents with an immigrant background as well as therapists in sectors with high load of immigrants would be one way to further study factors that decrease or increase help-seeking behaviours. If taboos and stigma related to mental health problems in general and of self-harm in particular are prevalent among those with immigrant background, one idea is to provide information regarding prevalence, detection, and treatment of such problems. Further, schools, various health services, and other relevant areas that are in contact with this population are of importance to identify possible barriers.

## Conclusions

This study reveals that the majority of adolescents with self-harm had not had any contact with CAPS, and among the group of self-harming adolescents with both SA and NSSH, only one in three had had such contact. While their CAPS contact was more prevalent with increasing psychosocial problem load, non-Western immigrant background appeared to be an important barrier. Better assessment of contact barriers and lowering the threshold for contact with CAPS among suicidal and self-harming youths are therefore important.

## Abbreviations

SH: Self-harm; NSSH: Non-suicidal self-harm; SA: Suicide attempt; CAPS: Child and adolescent psychiatric services.

## Competing interests

The authors have no competing interests.

## Authors’ contributions

All authors have contributed substantially to the manuscript. LM, IR and AJT participated in the design of the study, AJT drafted and revised the manuscript and IR, LM and EM made substantial contributions in revising it. AJT, IR and EM were involved in running the statistical analysis, and all authors were involved in interpretation of the analyses. All authors were involved in the interpretation of data. All have read and approved the final manuscript.
